# Edible Insects in Latin America: A Sustainable Alternative for Our Food Security

**DOI:** 10.3389/fnut.2022.904812

**Published:** 2022-05-27

**Authors:** Silvana Abril, Mariana Pinzón, María Hernández-Carrión, Andrea del Pilar Sánchez-Camargo

**Affiliations:** Department of Chemical and Food Engineering, Grupo de Diseño de Productos y Procesos (GDPP), Universidad de Los Andes, Bogotá, Colombia

**Keywords:** food production, eco-friendly, beneficial nutrition, Latin America, insects, entomophagy

## Abstract

Nowadays, the food industry faces paramount challenges in different areas, since worldwide consumers are increasing every day, and at the same time, they are demanding new convenient products. Recent studies show that the current food production system is unsustainable over time and therefore is necessary to create new alternatives of production. New food trends are focused on the consumption of natural products, that have an eco-friendly production approach, and a beneficial nutritional profile for the consumer’s health. Hence, products are being created to not only have good organoleptic characteristics, but also to contain a wide variety of micro and macronutrients, and to be sustainable within their production. For this reason, the use of raw materials that satisfy the needs previously mentioned is being implemented. For instance, the use of insects as raw material, because they have a high protein content comparable to animal-based foods. Specifically, ants and crickets can contain between 9 and 77% protein of dry weight, while beef contains between 25 and 28%. On the other hand, insects present an ease and sustainable production system, compared to livestock farming, since some of them feed with food waste generated by humans. In addition, require less food for their upbringing; insects can convert 2 kg of feed into 1 kg of insect mass, while cattle use 8 kg of feed to produce 1 kg of body weight. On the other hand, there is evidence that insects produce fewer greenhouse gases during their production, for example, pigs produce between 10 and 100 times more greenhouse gases per kg of weight. United States, Mexico, Chile, Peru, and Argentina have begun to develop and consume these products; thus, promoting different and new ventures. Large-scale production of insect-based food products could help solve or even prevent the looming food problem and contribute to the sustainable development goals set by the United Nations. Thus, the aim of this review work was to compile and investigate the edible insect’s alternatives in Latin America, as well as the commercially available or potential derivative products. We discussed the nutritional value of edible insects, and how they could contribute to food security.

## Introduction

**FIGURE 1:**
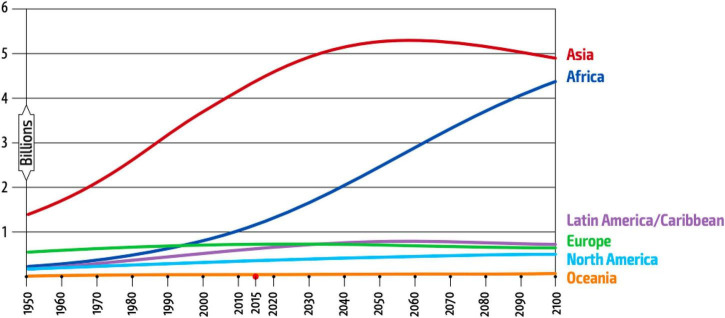
Population growth estimated by de FAO until year 2100 (7).

The constant growth of the population results in an increase of food production and consumption; it is estimated that by the year 2050, there will be approximately 10,000 million people (1). Figure 1 shows the population growth estimated by the FAO until the year 2100. Feeding this future population is one of the greatest challenges for agrifood industries, since not only demographic and economic factors must be taken into account, but also those related to natural resources such as water and energy (2). In countries like China, much of this population growth has occurred, and an increase in meat consumption has been associated (3). It is expected that by 2035 the population in China will be almost 1.41 billion (4), this rise in meat consumption brings China to surpass the US as the world’s leader in meat consumption (5). In China, meat consumption increased from 8 million tons in 1978 to about 9 million in 2020 (6); it is estimated that the global consumption of animal protein by 2050 could increase by 60% (7). These trends are facing several challenges for food and agriculture industries because meat consumption tendencies are causing massive deforestation, water scarcity, soil depletion and high levels of greenhouse gas emissions that cannot offer or promote sustainable agriculture and food production (8). According to a FAO report, published in 2017, if climate change and the exploitation of natural resources continue to intensify in the same way, humanity is at risk of not being able to feed itself (7). Livestock (including cattle, pigs, and sheep) occupies 70% of agricultural land, which corresponds to 30% of the land area and additionally 77 million tons of protein are used, of which 58 are recovered for human consumption (9). Likewise, livestock is responsible for 18% of all greenhouse gas (GHG) emissions of anthropic origin; the main sources and types of GHG from livestock systems are methane production from animals (25%), carbon dioxide (CO_2_) from land use and its changes (32%), and nitrous oxide (N_2_O) from manure and slurry management (31%). These gases are usually converted to units of CO_2_ equivalent (CO_2_ eq.) that have varying global warming potential (10).

This raises the question not only of whether it is possible to supply this growing demand, but also of the effects it would have on natural resources and the environment if this food production system is maintained (7). All these factors are presented as an obstacle to produce the food required by the population and animals (7). Therefore, it is necessary to search for alternatives that satisfy the future demand of food (11). Considering the above, in recent years, industries and some consumers are looking for alternatives to mitigate this effect. The demand for food is therefore changing toward new habits of production, sale, purchase, and consumption of food that are healthier, friendly to the environment and seek animal welfare (7). For this reason, innovative systems are needed to protect natural resources without reducing productivity. It becomes essential to make better use of resources where the production, conservation and packaging processes generate the least impact on the environment and ecosystems. Consequently, the models would be more sustainable over time and are going to be inspired by the circular economy (12).

Within this new system, the principle of efficiency is implemented, where the aim is to do more with less, that is, to develop functional foods, new ingredients and materials or energy from co-products, by-products, or waste (13). For example, waste such as bones, hooves, viscera, horns, skins, blood, feathers, bird carcasses, eggshells, among others, which have a significant content of protein, lipid compounds, fiber, etc., can be used as ingredients for food production or used in industry as natural additives (14). On the one hand, foods that are efficient in their production are also of interest because they require less space, water, and are less polluting. Likewise, the ingredients obtained from microalgae, bacteria, yeasts, fungi, or cultured animal cells are highlighted because they consume fewer natural resources (12). On the other hand, among the new foods in development is cultured meat or *in vitro* meat that is obtained from stem cells of animal muscles because it consumes 45% less energy, produces 96% fewer emissions and requires 99% less land (15). Similarly, insects have stood out in this group because they have high nutritional quality while generating environmental benefits (16). For these reasons, the breeding, production, distribution, and sale of insects, for animal and human consumption, started to be implemented a few years ago (17). Thus, the consumption of insects emerges as a possible solution for feeding an increasingly growing population and as an alternative to contribute to environmental sustainability.

In this way, the novelty of the present review was to identify and study the alternatives Latin America can offer regarding edible insects and how we could take advantage of them in order to develop products with the possibility to be commercialized. Taking into account the nutritional values these insects can provide us, we debated about its contribution to food security.

For this review article, databases such as ScienceDirect, Wiley, Web of science, Springer, and Researchgate, were used. To search for information related to the topic that the article is going to deal with, keywords such as *food security, edible insects, Latin America, sustainability, entomophagy, insects nutrition value, food safety, insects breeding, products based on edible insects, food of the future, insects rearing, insects*’ *consumption*, were implemented. Research was made not only of articles but also of web pages belonging to brands that work with edible insects, and books focused on the consumption of insects from the development of new products. It is important to mention that the period implemented when searching for articles, books, web pages, etc., was between 2000 and 2022.

## Entomophagy and Environmental, Nutritional, and Social Benefits of Insect’s Consumption

**FIGURE 2:**
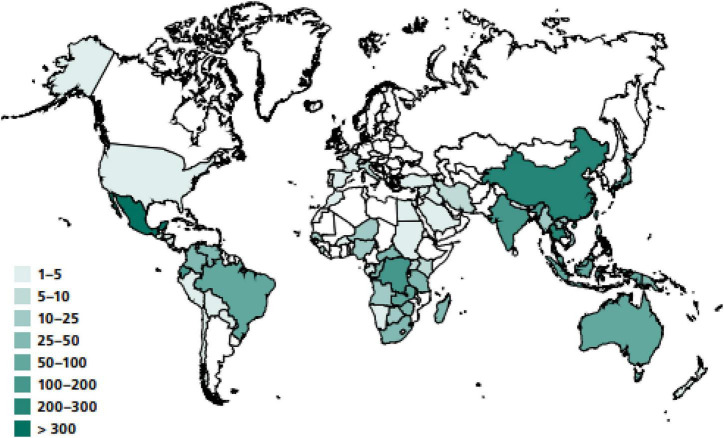
Recorded number of edible insect’s species, by country (16).

The term entomophagy refers to the ingestion of insects and has been considered an alternative or additional source of animal protein, which is an important source of micro and macronutrients for humans (18). Edible insect are estimated to be part of the diet of at least two billion people around the world, with more than 2,100 species of insects currently used as food (19). However, possibly all species of insect are edible (20). The most consumed insects worldwide are beetles (31%), caterpillars (17%) and bees, wasps and ants (15%). In addition, grasshoppers, crickets, locusts (14%), cicadas, leafhoppers, mealybugs, bedbugs (11%), termites (3%), dragonflies, flies, and others (9%), etc. are also consumed (21). Insects can be consumed at all stages of their development, including eggs, larvae, nymphs, pupae, and adults (20). They are raised, traded and exported in different ways: canned, fried, in syrup, with chocolate, with garlic sauce, among other preparations (22). In 2022 it was reported that entomophagy is practiced mostly in countries such as Thailand, Ghana, Mexico, China, Brazil, Australia, Japan, and the Netherlands (23). For example, China has a lot of edible insects but the two most famous ones are the *Antheraea pernyi* and *Bombyx mori* worms, the former was evaluated as a highly potentially edible insect (24, 25) and the latter as the perfect mass production system reared (26). Figure 2 presents a geographical map indicating the registry of insect species that are consumed by country (17).

However, despite various environmental, nutritional, and social benefits of using insects in the human diet, in Western countries most of the population rejects the idea of consuming them, for cultural reasons and for considering them unpleasant and harmful (27). Other aspects that cast doubt on the use of insects to feed animals and humans is the legislative issue and whether they can really be produced on a large scale to meet the high demand (28). Nevertheless, entomophagy is a practice to which benefits are attributed in different fields such as the environment, consumption and social, as discussed below.

### Environmental Benefits

Due to insect are cold-blooded species, food conversion becomes really efficient compared to other species. There is an evidence that insects emit less greenhouse gases and ammonia compared to conventional livestock (20, 29). Thanks of these benefits, insect farming has been suggested as a promising alternative to conventional livestock production, even in Western societies (30). Another environmental advantage of this feeding practice is the use of food waste as feed for insect farms (17). First, various insects feed on food waste that humans and traditional livestock cannot eat. Insects eat food waste and as a result, a bad disposition and incineration are avoided (17). Likewise, insects do not compete with humans for the same food, as cattle do in some cases, that is, insect farming does not take from the planet’s already limited resources, but rather helps reduce food waste (31, 32). In the same way, entomophagy is a practice that uses much less water than traditional cattle since there are insects such as mealworms that are more resistant to drought (17). Finally, insect are less dependent on the land than traditional livestock farming since their small size allows them to take more advantage of the ground space both horizontally and vertically (29).

### Consumption Benefits

Insect are being cataloged as a food source of high nutritional value, compared to meat or fish, since some of them have a large amount of micro and macronutrients (33). This food especially contains proteins, amino acids and is rich in fiber and minerals such as copper, iron, magnesium, phosphorus, manganese, selenium, and zinc (34). Proteins represent the main component in the nutritional composition of insects and their content is variable. Beetles and larvae have a protein content between 20 and 71%, flies and mosquitoes between 36 and 70%, dragonflies between 37 and 68%, bees, bumblebees, wasps, and ants between 10 and 64%, caterpillars and moths between 13 and 64% and crickets and grasshoppers between 27 and 76% (20). Different kinds of insects have a high protein content that is comparable to foods derived from animals such as beef (25–28%), pork (20–37%), chicken (22.8%) (35, 36), and fish (14–63%) (22). Similarly, insects have good quality amino acids and are rich in essential amino acids. The main amino acids they contain are glutamic and aspartic acids, phenylalanine, alanine, proline, leucine, tyrosine, valine, and methionine (20). Likewise, edible insects have registered high digestibility values, although the species of insects and the technological processing can influence this parameter (17). It is worth noting that digestibility of insect proteins is between 78 and 98% (20).

As it can be seen, high protein content, which includes all essential amino acids, the presence of unsaturated fatty acids, vitamins, and minerals, make both insects and meat highly nutritious (33). Although there is still extensive research required on the nutrient composition of edible insects, it is undisputed that they represent an alternative sustainable source for animal-related protein for food and feed in general (37).

### Social Benefits

In terms of benefits for the social environment, important livelihood diversification strategies can be achieved through the breeding and collection of insects. This is because they can be collected directly from the environment in a simple way and technical means or significant investments are not required to access basic breeding and collection equipment (38). On the other hand, catching, cultivation, processing and sale of insect are activities that can contribute to a direct improvement of the diet and income for society in general (39). Likewise, the breeding and collection of insects can generate business opportunities in developed economies. Lastly, these animals can be easily processed to serve as food for humans as some species can be consumed whole or can be made into paste or ground to make flour or extract their proteins (17). Considering these characteristics, the economic and social factors that make insect farming more desirable than animal husbandry are that insect harvesting and breeding require low technology and capital investment choices and that the cultivation of insects provides living opportunities to both the urban and the rural population (40, 41). Additionally, due to the high resource efficiency and good nutritional value of insects, insect rearing for entomophagy seems to fit perfectly with a modern food production system (30).

## Types of Edible Insects Around the World and Latin-America

**FIGURE 3:**
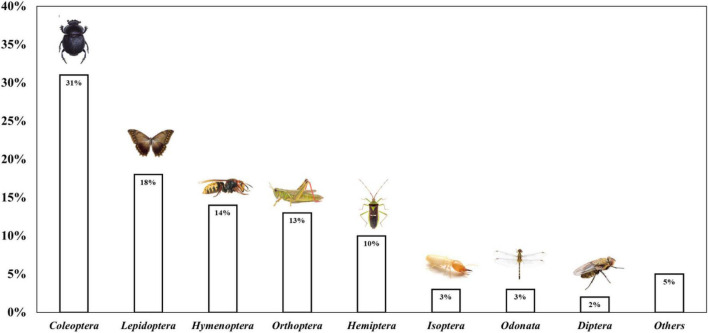
Orders of species of insects that are more consumed (46).

**FIGURE 4:**
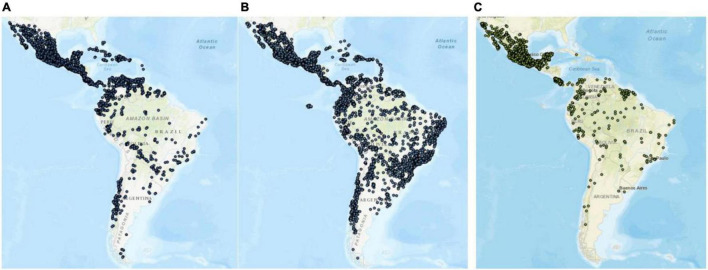
Latin America’s insects. **(A)** *Coleoptera*’*s* in Latin America (42), **(B)** *Hymenoptera*’*s* in Latin America (45). **(C)** *Lepidoptera*’*s* in Latin America (43).

In many parts of the world, especially in the middle east, insect are commonly used as a source of food. As of 2012, over 1,900–2,100 species have been recorded as part of the diet in continents as Asia, Africa, Oceania, and North and South America (38). This is because of the high diversity of insects that are highly nutritious and a healthy food for people, but it is also because of the ability of many species to breed quickly. Additionally, insect are efficient in converting their food into protein, and some species can be reared on organic waste. As it was said, the orders of species of insects, that are most consumed are the ones shown in Figure 3. *Coleoptera* is the largest order as it has 350,000 species that are found in almost all habitats. Nowadays, Mexico, Costa Rica, Venezuela, Colombia, Ecuador, and Chile are Latin American countries that have more species belonging to this order according to Data Basin: a science-based mapping and analysis platform of environmental stewardship (42). *Lepidoptera*, the second largest order of insects, includes the moths, butterflies, and skippers, and there are approximately 180,000 species. In Latin America, *Lepidoptera*’*s* are more common in Mexico and Costa Rica (43). *Hymenoptera* includes such familiar groups as ants, wasps, and bees; these insect are consumed in many regions of the world because of the nutritional value they have (44). The countries in Latin America that have a lot of species belonging to this order are Mexico, Costa Rica, Panama, Colombia, Ecuador, and Brazil (45). The *Orthoptera* group includes grasshoppers, locusts, katydids, and crickets; common edible insects worldwide, particularly in Japan and Thailand (38). The *Isoptera* group are insects that live in nests of diverse morphologies that receive the name of termite mounds. At last, *Odonata* and *Diptera* are the orders that mostly incorporate dragonflies and flies, respectively. Dragonfly nymphs are an accepted food in many regions of the world, with 29 edible species recorded. On the contrary, flies and cockroaches are not typical foods for people, although both types of insects were eaten in ancient times. Different species of flies that feed on organic matter can be used to convert organic wastes into fertilizers. Moreover, the bodies of insect are rich in protein and are therefore used as animal feed (46). In 2014, 735 species of insects were scientifically identified in Latin America. Table 1 shows the number of edible insect species in the different countries of Latin America (47). These countries consume more *Coleoptera* species, then *Hymenoptera*, then *Lepidoptera* and finally *Isoptera*. Likewise, it was identified that northern Mexico, Colombia, Ecuador, Venezuela, and southern Brazil are the countries that consume and use the most insects for food. In Figure 4 can be observed that Mexico and Costa Rica have more of those groups of insects in Latin America compared to the other countries (45).

**TABLE 1:** Number of edible insects in different countries of Latin America [modified from Medeiros (47)].

Country	Number of species	Percentage (%)
Mexico	415	56.5
Brazil	122	16.6
Ecuador	78	10.6
Colombia	51	6.9
Venezuela	39	5.3
West Indies	6	0.8
Guyana	4	0.5
Nicaragua	4	0.5
Peru	3	0.4
Chile	3	0.4
Paraguay	2	0.3
Bolivia	1	0.1
Honduras	1	0.1
Panama	1	0.1
Jamaica	1	0.1
Guatemala	1	0.1
Suriname	1	0.1
Trinidad	1	0.1
Barbados	1	0.1
Total	735	100.0

## Nutritional Properties and Quality of Edible Insects

The nutritional values of insects can vary depending on their metamorphosis, origin, diet, and the preparation processes carried out before the consumption (48). In addition, the composition of insects is also different depending on the species to which the insect belongs (34). However, many of the edible insects provide enough energy and protein for the human diet and, furthermore, some are healthier than many of the meats that are consumed today. According to Payne et al. (49), crickets, palm weevil larvae and mealworm are significantly healthier than chicken and meat. Finally, it is important to mention that insects also contain enough energy, protein, amino acids necessary for the human diet, and have a high content of mono and polyunsaturated fatty acids and vitamins (48). Nutritional data indicates that the content of good quality protein is high in insects, since it is approximately 50% of the total weight of the insect and, in addition, that they contain high contents of essential amino acids (46). In Table 2, the proximal composition of some orders is shown. *Ortodopthera* stands out for its high protein content (61.32 g/100 g db). Into the detail, Table 3 compares protein content between some edible insects and foods derived from animals. Also, it is important to mention that some insects store large amounts of fat; approximately between 10 and 60%. The insect that has the greatest fat content is the dragonfly, since it has between 8.6 and 15.2 g per 100 g of insect (48). In addition, generally they have high levels of unsaturated fatty acids. For example, termites, belonging to the *Isoptera* order, have a proportion of 74.19% unsaturated fatty acids, ants have 79.99% unsaturated fatty acids and grasshoppers a value of 77.5% unsaturated fatty acids (46). Regards to the fiber content, it is between 5.06% for *Isoptera* to 13.56% for *Hemiptera*. Species with a lower fiber content are part of the orders *Lepidoptera*, *Hymenoptera* and *Orthoptera*; *Aegiale hesperiaris*, *Apis mellifera*, and *Brachytrupes* spp., respectively, since they contain between 0.12 and 11.61% of fiber (50). Similarly, insects have good quality amino acids and are rich in essential amino acids; the main amino acids they contain are glutamic and aspartic acids, phenylalanine, alanine, proline, leucine, tyrosine, valine, and methionine. In addition, glutamic acid, leucine, and alanine are also highlighted (20). The amino acid that orders contain the most is leucine; 74.2 mg/g protein for *Coleoptera*, 62.7 mg/g protein for *Lepidoptera*, 78.4 mg/g protein for *Hymenoptera*, 74.8 mg/g protein for *Orthoptera*, 49.8 mg/g protein for *Hemiptera*, 78.3 mg/g protein for *Isoptera* and 57.4 mg/g protein for *Diptera* (50).

**TABLE 2:** Proximal composition of some orders [taken from Rumpold and Schlüter (50)].

Order	Protein* (%)	Fat*(%)	Ash* (%)	Fiber* (%)	MUFA (%)	Energy content* (kcal/100 g)
*Coleoptera*	40.69	33.40	5.07	10.74	35.72	490.30
*Lepidoptera*	45.38	27.66	4.51	6.60	23.36	508.89
*Hymenoptera*	46.47	25.09	3.51	5.71	48.76	484.45
*Orthoptera*	61.32	13.41	3.85	9.55	29.37	426.25
*Hemiptera*	48.33	30.26	5.03	12.40	32.39	478.99
*Isoptera*	35.34	32.74	5.88	5.06	22.00	508.89
*Odonata*	55.23	19.83	8.53	11.79	NR	431.33
*Diptera*	49.48	22.75	10.31	13.56	47.23	409.78

*Results based on 100 g of dry basis.

*MUFA, Monounsaturated fatty acids; NR, Non-reported.*

**TABLE 3:** Protein content of edible insects and foods derivates from animals (50).

Edible insects	Protein (%)
Beetles	20–71
Flies and mosquitos	36–70
Dragonflies	37–68
Bees, bumblebees, wasps, and ants	10–64
Caterpillar and moths	13–64
Crickets and grasshoppers	27–76
Beef	25–28
Pork	20–37
Chicken	20
Fish	14–63

*Pork (35), fish (22), and beef and pork (17).*

As it can be seen, protein, fat, mineral and vitamin, essential components to sustain life, contents in insects generally satisfy the requirements of healthy food, although there is considerable variation associated with insect species, collection site, processing method, insect life stage, rearing technology and insect feed (51, 52). In the same way, edible insects have the potential to benefit human health due to their nutritional characteristics (53). For instance, some insect-based food, like cricket powder, contain high quantities of bioactive peptides with antioxidant and antimicrobial properties (53). Also, the unsaturated fatty acid content of insects may help to reduce the risk of developing cardiovascular disease (54). Moreover, contents of essential micronutrients in crickets and other edible insects have the potential to prevent anemia and improve immune function, cognitive function, and gastrointestinal health in humans (53). The acquired knowledge indicates that edible insect are a valuable food product and that their widespread use in the human diet may help solve the problem of global malnutrition (33).

Even though insect are not generally consumed due to lack of knowledge in taste and little familiarity, there are countries that have a remarkable market value for edible insects and consume them because of their high nutritional quality. For instance, Latin America, with a market value of $92.2 million in 2018, is the second largest market for edible insects in the world. Additionally, it is known that Mexico, Ecuador, Colombia, Brazil and Venezuela, consume the most insects as an alternative food source (55). It is important to know that the total production around the world of edible insects per year is 60,000–67,000 tons approximately of which 4,500–6,000 are produced in Latin America. In Mexico it is common to consume the following insects: “Chapulines” (grasshopper), “Chicatana” (ant), “Gusano blanco maguey” (maguey grub), “Escamol” (ant egg), and “Ahuahutles” (mosquito eggs). It should be noted that ants or “Chicatanas” are also consumed in countries such as Colombia and Brazil. The nutritional composition and essential amino acid content of these five species are shown in Table 4 (56). Likewise, it is important to mention that the beetle, belonging to the Coleoptera order, is in the ranking of the most consumed insects in Latin America (55). Specifically, the most famous beetle, which is widely produced in Latin American and Caribbean countries, is the *Tenebrio molitor*, commonly known as the mealworm or flour beetle (57). Optiprot, one of the many Mexican companies that work with this insect, produces 0.4 tons of this edible insect per month, and that is, 4.8 tons of mealworm per year (57, 58).

**TABLE 4:** Nutritional composition of five edible insects from México (g/100 g dry basis) (56).

Insect/component	Chapulines	Chicatana	Gusano blanco maguey	Escamol	Ahuahutle
Protein	71.50	66.00	30.80	40.90	53.60
Lipids	5.75	24.02	52.55	33.96	4.33
Minerals	2.50	3.00	2.29	7.85	21.00
Fiber	3.89	2.06	0.12	1.30	3.00
Isoleucine	4.20	5.10	4.50	4.50	5.00
Leucine	8.50	7.50	6.10	7.60	8.00
Lysine	5.70	5.10	5.00	5.50	3.50
Methionine	4.30	4.00	3.10	4.50	2.90
Phenylalanine	7.70	7.50	7.00	6.60	6.20
Threonine	3.90	4.10	4.10	4.30	4.00
Tryptophan	0.60	0.60	0.80	0.70	1.10
Valine	5.60	6.00	5.10	6.00	6.00

## Insect Production Systems and their Derived Products

**FIGURE 5:**
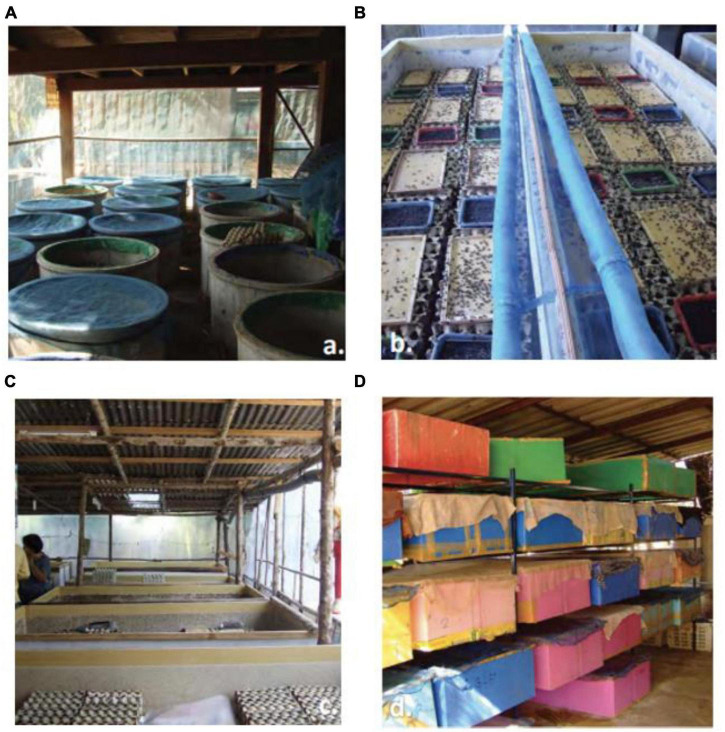
Types of breeding Containers for Crickets. **(A)** Concrete cylinder pen breeding container, **(B)** concrete block pen breeding container, **(C)** plywood boxes breeding container, and **(D)** plastic drawers breeding container (67).

The rearing of insects has been practiced for 7,000 years with different purposes like sericulture (silk), apiculture (honey), biological control of pests and the production of medicinal products (59). It has deep roots in tropical areas of Asia, Africa, and Latin America, where insects represent an important component of local diet. However, the practice has progressively expanded to Western countries (60). For insects to be considered a viable micro livestock, it must be possible to produce them on a large scale in a sustainable, secure and qualified way (17). Until now, they have developed controlled and artificial conditions for mass rearing. However, there are still several hurdles preventing the scaling up of insect farming for human and animal consumption (60, 61). The most important barrier is that until now there is not known the ideal specie of insect that have high egg production, a short larval stage, high weights of larvae or pupae, a high productivity, low feed costs, low vulnerability to diseases, ability to live in high densities, and a high-quality protein content, among others (61). In the same way, optimal conditions of temperature, light, humidity, ventilation, feed composition and quality, etc., are not yet known (62). The efficiency of insect farms in terms of feed conversion depends on rearing conditions. Temperature and humidity must be ensured to optimize the insect growth; typically, temperatures range between 20 and 35^°^C and humidity levels from 55 to 75% are required (60, 63). For large-scale production, critical elements including research on insect biology, suitable rearing conditions, and diet formulas are required (64). Moreover, nowadays for insect farming it is necessary manual labor to complete tasks such as feeding, collection, cleaning and rehousing which has as a consequence that farm-reared insect are expensive, even when feed costs are low (61). This means that to achieve commercial mass production automation is needed for insects to be an attractive alternative and economically competitive to beef and poultry protein (61, 64). Rearing insects in mechanized facilities involves high designed equipment, mechanization, industrial intensive, varieties of production elements, computerizations and cost effectiveness (65). To produce insects, two units are needed: (i) a reproduction unit where adults can mate and lay their eggs and (ii) a production unit where the eggs are sown on substrate (66). In Figure 5 can be seen different types of breeding containers (17).

Nowadays, there is no ideal insect production system for most kinds of insects (68). The industrial production scheme of insects is based on controlling the life cycle of the target species of production, being separated, like other livestock productions, by stages of reproduction, rearing and fattening (69). Dobermann et al. (61) proposed a general model for insect production with four different phases. First, it is important to consider the specific feeding, for the type of insect, and in this way begin the rearing process [larvae/nymph, pupae, adult (breeding colony), and finally eggs]. It is fundamental to highlight that at this stage, special care must be taken with the conditions for raising the insect and it is essential to monitor the quality and safety of the crop. Secondly, classification of the different stages of development of the insects will proceed and then the automated harvesting process. For instance, adults are kept in containers that are provided with feeding substrate and water (60, 70). In the same way, oviposition sites should be reduced to specific locations inside the breeding containers to allow easier egg collection. Small boxes sealed with moisture can be used where females can preferentially spawn. Decomposing grain saturated with water, peat moss, coconut husks and other organic matter can be also used depending on the insect species (60). In third place, the post-harvest process of insects is proposed, which includes the process that would ensure food safety and quality and the processing of the insect into an edible food (61). Finally, the last phase consists in the packing, storage, transportation and consumption of food or feed (61). Nonetheless, in some countries the mass farming of specific types of insects already exists. For example, crickets are grown in large boxes ranging from 36 to 60 cm deep filled with different materials, most commonly cardboard, such as egg cartons (Figure 5C) or packing dividers for shipping to increase surface spaces (Figure 5B) (71). Additionally, these insects should be reared in humid environments and for that reason they are traditionally raised in plastic bins that contain damp sponges (to maintain the humid environment) (72). Favorable conditions for crickets are warm temperature (29–35°C) and high relative humidity (50%) (73, 74). Additionally, on the bottom of the box they must have a layer of potting soil or something similar. On the other hand, a female cricket usually lays around 100 eggs, but it is possible that over the course of her life (8–10 weeks as an adult) she lays 3,000 eggs (72). The eggs hatch in about 6–14 days but this will depend with the temperature and cricket species. At temperatures of between 28 and 35°C, the eggs hatch between 1 and 2 weeks. At low temperature eggs take a longer period to hatch, 20–21 days at 25°C and 76 days at 15°C (75). Each harvesting cycle is between 28 and 35 days (76). In the United States, Canada, and Europe, crickets are typically fed a combination of vegetables, corn and soybeans (77). However, kales (*Brassica oleracea var. ocephala*), banana peels (*Musa acuminate*), sweet potato vines (*Ipomoea batatas*), and ugali are used as cricket feed (78). According to a United States industry consultant, crickets are slaughtered by different methods; by freezing, shredding or heating. Sometimes, they freeze-dried alive or dead to remove all water (72). On the other hand, unlike crickets, the first stage of mealworm farming is breeding darkling beetles (79). Female beetles lay approximately 500 eggs during her lifetime and they will take about 2–3 weeks to hatch (80). After eggs hatch, producers focus on growing the larvae as quickly as possible; they will take about 10–12 weeks to reach the right size (80). Mealworms usually use diets of cereal bran or flour and fresh vegetables (e.g., carrot, apple, potato, or cabbage) as a water source, or flour with water and a source of protein. Protein sources commonly used to complement the diet include beer yeast, casein and soy protein (81, 82). At this moment, they are separated from the colony and they are then slaughtered in a variety of methods; the most common methods are freezing or freeze-drying (72). Finally, mealworms can adapt to tolerate a wide range of temperatures, as they act and eat normally at 15–40°C, and can survive at 0–15°C and 40–45°C (83) and they must have 60–75% relative humidity (82). Black soldier flies originally came from Latin America, but are now established in most of the world, and are common in the United States and Europe (84). The rearing of these insects requires hot and humid conditions and typically, they are raised in netted. The best range of temperature for the larvae to pupate is from 25 to 30°C. For mating purposes, optimal temperature is around 28°C (85). According to Bullock et al. (86), black soldier fly larvae develop most rapidly at 70% humidity boxes. Usually, they are feed with cereal, fruits and vegetables that are positioned at the bottom of the box (72). Black soldier flies typically lay eggs in cracks and crevices and these eggs must be moved and maintained in a nursery bin with high temperature, up to 30^°^C; they hatch after up to 10 days (87). The females lay between 320 and 1,000 eggs, on a dry substrate in a humid environment (87). On the other hand, the larvae of this insect might be an especially cheap insect to farm because they are usually fed with cereals, fruits, vegetables, meat and animal waste. Finally, when black soldier fly larvae are fully grown, they crawl out of the substrate; in other words, they “self-harvest.” This insect is frequently shredded and freeze-dried to produce a powdered product (74).

Taking into account that the breeding and reproduction of insects varies depending on their class, it is evident that their processing to turn them into food for humans or animals will also be different (88). New technologies are currently being developed to transform insects into edible foods that meet safety regulations (89). Pre-processing technologies represents the first step of each edible insects processing route and mainly consist of insect harvesting/separation from the substrate residuals, insects’ inactivation, removal of wings/legs, and washing (50). First, there is blanching, which is used to reduce microbiological contamination and inactivate enzymes. It consists in a short boiling step followed by a rapid cooling in flowing cold water to reduce microbial counts and to inactivate the enzymes responsible for spoilage and food poisoning (90). It is usually performed prior to food processes such as drying, frying, canning, and freeze- drying (91). However, the most used technology to increase insect’s life is drying, since it reduces microbiological activity, degradation reactions and moisture content (92). It is important to highlight that when carrying out this process, alterations in the nutritional and physical profile of the insect can occur like alterations in protein content, lipid oxidation, and color variation (93). Other methods are often used to ensure a better ratio of macronutrients in the final insect feed, e.g., to reduce lipid or chitin contents, or to obtain derived products such as oil and protein powders and pellets.

These processes have allowed edible insects to be marketed in different ways today; for example, whole (dried, frozen, and pre-cooked), processed or in extracts (94). In Western countries, the practice of entomophagy is still not very common because insect are associated and classified as unpleasant, dirty, and dangerous (17). However, in Colombia the consumption of insects is common despite being a westernized country (95). The Santandereana ant known as the “Culona” ant and whose scientific name is *Atta laevigata* is common in the departments of Santander and Casanare in Colombia. They are used as food by the inhabitants, attributing aphrodisiac properties to them (96). In 2013, in the department of Santander, Granados et al. (96) carried out on toasting and the production of flour from Santanderean ants. That article describes the different stages of pre-treatment and treatment to obtain ant toasted flour. In the first place, to fry or roast the ants, they must be collected, which is done outside the anthills manually or with the help of equipment, such as a sucker. The collected ants are transported in sealed containers to the place where the selection and cleaning is done. The future queen ants are selected, their wings, legs and head are removed and the residues of sand, stones, earth, etc. are removed. This selection operation is done manually. Afterward, a blanching process is carried out for 2 min at a temperature of 89.85°C until the ants are sacrificed. Subsequently, the elaboration of the roasted product begins. Firstly, pre-roasting is done for 30 min at a temperature of 69.85°C and then for toasting a temperature of 89.85°C is used for 20 min. Then, grinding is carried out where the aim is to reduce the size of the particles. Having the crushed product and there being a greater contact surface with the medium, a more even drying is achieved for 1 h. The final moisture percentage should not exceed 5%. Then the unitary sieving operation is carried out with a sieve of 40 microns in diameter to obtain a final product with uniform granulation. Finally, they are placed in the transport and commercialization containers (96).

Finally, it is possible to conclude that in order to scale the production process of insects and products derived from them, the breeding and growth characteristics of the species of insects must be known (97). Additionally, a good post-processing of the insects must be guaranteed so that they can be transformed into edible food (38). So far, in Eastern countries, specific production systems have been developed for some insects, such as crickets and flies, since their characteristics are already known (70). In the same way, these production systems already have their regulation (98). On the contrary, in Western countries, the practice of entomophagy is new and therefore there is not enough information to allow the implementation of a definitive production process (99). However, processing systems such as that of the “Culona” ant are beginning to be studied few years ago (96). Nevertheless, it can be highlighted that production systems of edible insects worldwide are trying to implement good manufacturing practices not only to warrant the processing of the insects into an edible food but also to guarantee food safety and quality (61). In western countries, providing information about the nutritional and environmental benefits of entomophagy is a common intervention aimed to increase Westerners’ acceptance of edible insects (100).

## Current Food Products that are Commercialized or With the Potential to Be Commercialized

There is a large number of ventures in the world, where the focus is insects as food for humans. In the last 10 years, around 133 companies from Europe, South Asia and North America have sold this type of food (20). For example, they sell different types of bread, pasta such as curry, salsa, candy for children, pate, energy bars, drinks with or without alcohol, cookies, corn tortillas, pasta, hamburgers, sausages, among others (20). Much content of these products is based on insect meal; amounts between 1 and 25%, with crickets being the most used in the first place, followed by fly larvae and mealworms (20). Some of the most recognized companies in the United States, Europe, and Mexico are: Exo Protein (United States), Entosense (United States), Brooklyn Bugs (United States), Entomo Farms (United States), Chapul (United States), Griyum (Mexico), BeCrickets (Mexico), Smart Bites (Mexico), Becrit (Spain), Crunchy Critters (United Kingdom), Entis Store (Finland) and Jimini’s (France). It is important to emphasize that each of these enterprises manages different products for different users. For example, “Exoprotein” manages cricket flour, energy and protein bars for athletes (101). “Becrickets” manages cricket flour as the only product and is also focused on people who practice sports due to the high amount of protein that this product has (102). On the other hand, “Becrit” is an enterprise that manages a single product, and this is known as an insect-based protein shake which has three different flavors: strawberries and white chocolate; vanilla, coconut, and cinnamon; and chocolate. It is a shake intended for those people who have an active physical activity but who specifically do crossfit (103).

One of the enterprises that manages the most products is “Crunchy Critters” since within its offer are buffalo worms, mealworms, crickets, and lobsters, with which they make all kinds of snacks or flours (104). On the other hand, “Entosense” is a venture that aims to bring insects closer to the American diet with the help of various products that adjust to their tastes, that is, salts, lollipops with insects inside, cookies, snacks, etc (105). A venture like “Entosense” is “Brooklyn Bugs,” however, the difference between these two is that “Brooklyn Bugs” does not sell products but rather conducts training or events where knowledge about their preparation is acquired.

It is necessary to highlight that there are ventures that point to the commitment to enhance the care of the environment. One of these enterprises is “Griyum” which distributes all kinds of products but from other companies and offers companies the opportunity to transform processed products in foods rich in nutrients (106). Another venture that has the same objective of improving the environmental situation is “Entomo Farms” which manages fertilizers, pet food, protein shakes, protein bars, snacks, cricket flour, among others (107). “Smart Bites,” “Chapul,” and “Jimini’s” are ventures that target people who care about their health through diet and exercise, are not afraid of eating insects and/or seek natural products over processed products (102).

On the other hand, several United States companies, as stated above, have become interested in the business of producing and selling insect food products. “Chirps Chips” make use of crickets from some North American farms and produce cricket “chips” that additionally contain corn, bean, and chia seeds, making them a product of nutritional interest (32). Similarly, other companies develop these kinds of products such as “All Things Bugs,” that produces and sells cricket powder; “Chapul,” which manufactures cricket protein bars (108); “Sens,” that produces cricket protein bars and cricket flour (109) and finally “Hotlix” that produces and sells candies based on insects such as ants, scorpions, crickets, and worms; one of its innovative products is the inclusion of foods such as chocolate covered crickets and scorpion lollipops (32).

Now, focusing more on food products based on insects made in countries from Latin America, it is important to highlight that most, or it could be said that almost all of them, do not have a regulatory framework that stables the breeding of insects for the production and marketing of their products (110). For example, Argentina does not have such a framework, however, progress has been made in the creation of one where the genuineness of insects and the establishment of criteria for their safety will be ensured (111). Companies such as “Ento Piruw” (Peru) and “Food for the future,” better known as “F4F,” (Chile) handle insect-based products but are aware that in their countries they are not regulations that establish the use of insects in human food (20). “Ento Piruw” is responsible for the production and marketing of energy bars made from the species *Tenebrio molitor* (mealworm) and its product is aimed at the sports sector (112). On the other hand, “F4F” is a Chilean company that will open a plant to produce insect meal and oil in order to help reduce the carbon and ecological footprint in salmon farming; they seek to ensure the food of the future (113).

**FIGURE 6:**
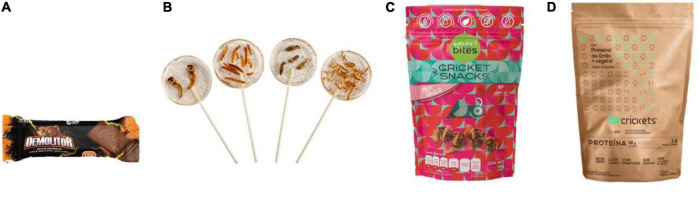
Food products derived from edible insects. **(A)** Demolitor’s protein bar ^®^ (Ento Piruw), **(B)** edible insect lollipops ^®^ (Crunchy Critters), **(C)** Cricket Snacks - Maca Canela ^®^ (Smart Bites), **(D)** Cricket Proteína + Vegetable Chocolate Flavor ^®^ (BeCrickets).

In countries like Brazil, not many insect-based products are known since their consumption is undervalued due to the few studies that exist about entomophagy (114). However, studies indicate that most Brazilian indigenous groups do tend to consume them (114); it is important to mention that in Latin America, there are approximately 50 million indigenous people and that is, about 10% of the total population (115). Back to the topic, 39 Brazilian indigenous groups, consider that insect are one of the most fundamental food resources in their diet and they can consume grasshoppers, termites, cockroaches and even their own lice (114). Another of the Latin American countries that does not have a specific regulation to produce insects is Costa Rica, however, it is recognized for being a tropical country with great capacities to produce crickets. Costa Rica can produce a large amount of flour based on crickets (116). Statistics indicate that it is expected to produce and export an amount of 2 tons of cricket flour to countries such as Mexico, the United States and some European countries in the year 2024 (116). In Figure 6, can be seen some of the products mentioned above.

Returning to the topic, there are companies that have goals such as: perfecting insect-based products and expanding traditional gastronomic diversity. “Arthrofood,” is a Colombian company, which in addition to fulfilling the two aforementioned objectives, seeks to save the world from our table and have a future with sustainable food (117). It is a company that breeding two species of tropical crickets: *G. assimilis* and *G. sigillatus*; through mating, incubation of eggs, to finally produce flour from this insect; they implement a totally friendly alternative to the environment (118). At the same time, the consumption of insects in Colombia is given in greater proportion by the indigenous people belonging to the Amazon basin. For example, indigenous groups like Yagua, Bora, Iquitos, and Capicuna (119), consume in their daily diet, as a source of protein and fat, the larva of the beetle *Rhynchophorus palmarum* (120). Today not only the indigenous people consume it, but also restaurants use it as a raw material. For example, in Medellín Colombia, the restaurant inspired by Amazonian flavors: “La Chagra,” includes a dish whose recipe is focused on these larvae and is known as the “Pachamama” dish (118). In the department of Santander, another species of insect known as the “culonas ants” (*Atta laevigata*) are consumed, which have a very high commercial potential and are also very well received in the market to develop products based on it (119).

Although this alternative is not widely used in Colombia, there are studies focused on the possibility and formalization of new insect-based products that demonstrate the nutritional and sensory potential of entomophagy. For example, students from the Universidad de los Andes, developed the formulation of a hamburger meat based on cricket flour and subsequently studied the properties and microbiological quality of the product. This study proposed an experimental design in which the percentage of cricket flour and the type of binder used in the formulation were varied. As a result, they found that the best formulation to resemble their control sample: Pietran’s Veggie meat; was using 25% cricket flour and potato starch (121). On the other hand, there is a study, where a granola-type bar based on cricket flour was developed. This bar not only met attributes similar to those of an ideal bar, but was also known as good protein source since it provides 16.76% of the recommended daily value. Its percentage of total carbohydrates was lower than that of commercial bars and its percentage of moisture complied with the standard (95). There are also books that provide information and recipes for the development of products or dishes from insect-based ingredients. A specific case would be the book “Desde Cundinamarca. Harina de grillo: gastronomía y sostenibilidad para Colombia y el mundo,” which has a large number of recipes that allow the reader to discover different ways of implementing cricket flour and at the same time realize how it can contribute to the development of a healthy and sustainable diet (122).

## How Can Insects Contribute to Food Security?

Food security is a problem in many developing and less developed countries due to increase in human population and decrease in crop productivity and food availability (40). Food security exists when all people, at all times, have physical and economic access to sufficient, safe and nutritious food that meets their dietary needs and food preferences for an active and healthy life (89). The four pillars of food security are availability, access, utilization and stability; while the nutritional dimension is considered integral to the concept of food security (123). Nations worldwide are feeling increasing pressure to improve the food system and to overcome the food crisis. For that reason, companies and governmental agencies have implemented various methods to improve food supply by introducing new technologies that are more efficient, cost-effective, and yield better crops such as genetically modified foods (124). However, food insecurity is still a common problem among low-income households in developing countries (125, 126).

The Sustainable Development Goals (SDGs) aim to end all forms of hunger and malnutrition by 2030, making sure all people—especially children and the more vulnerable people—have access to sufficient and nutritious food throughout the year (127). The aim is to ensure that everyone everywhere has enough good-quality food to lead a healthy life. This involves promoting sustainable agricultural practices improving the livelihoods and capacities of small scale farmers, allowing equal access to land, technology and markets (128). It also requires international cooperation to ensure investment in infrastructure and technology to improve agricultural productivity (127). The data for Latin America indicate that 187 million people suffer from moderate or severe food insecurity. Of these, 53.7 million were in a situation of severe food insecurity during the same 3-year-period. Approximately two-thirds of that population was concentrated in South America, and the rest in Mesoamerica (129). In South America the prevalence of moderate or severe food insecurity increased by 20.5% between 2014 and 2020, while in Mesoamerica there was an increase of 7.3% during the same period (130). The Food and Agriculture Organization (FAO) of the United Nations took an initiative to create a policy and proposed the program of feeding people with alternative sources which includes insects (38). The consumption of insects has been gaining popularity in many Western countries as an environmentally friendly alternative to conventional proteins such as chicken, beef and pork. There are already several companies producing insects for human consumption (131) due to the facility in their rearing, short life cycle, high intrinsic growth rate, the good nutritional properties and the benefits on the environment (132).

Apart from the reasons mentioned above, insects have been well-recognized worldwide as nutritious food since insects provide proteins (amino acids including methionine, cysteine, lysine, and threonine), carbohydrates, fats, some minerals and vitamins, and have energy value (133). In tropical countries most insect species are collected from nature. The reason that insect are predominantly eaten in tropical countries is that they are larger and often occur clumped, which facilitates harvesting. Also, in the absence of a winter season, insect species can be found during the whole year (123). However, consumer disgust remains one of the largest barriers to the adoption of insects as viable sources of protein in many Western countries.

It has been demonstrated the feasibility of the obscure incorporation and fortification of a major food staple, such as rice, by utilizing edible insect flour as a value-adding ingredient. By removing the original form of the insect and incorporating its flour into a food matrix that is widely consumed around the world, could provide an innovative solution for food insecurity and malnutrition, especially in developing countries. Furthermore, the hidden form of the edible insect is designed to reduce the “yuck” factor and increase the acceptance of entomophagy (134). Edible insects, with their high feed conversion efficiency and fecundity, as well as their minimal space for rearing, certainly represent as an advantageous solution for present and future food insecurity (134).

As a conclusion, it is evident that inefficiencies in the current food production system generate inconsistencies between the demand and supply of food resources. The animal protein is not evenly distributed across the globe, as the average person in a “developed” country consumes 40 g more protein a day than the average person in a “developing” country (9). The demand for affordable and sustainable protein is high, while animal protein is becoming more expensive and less accessible in some regions (70). Edible insects have been suggested to be capable of providing a valuable source of food in geographical regions with people suffering from malnutrition and food insecurity, since they can provide protein, vitamins and minerals needed for human health and wellbeing (38, 135). Additionally, entomophagy may be a viable solution to the problem of global food insecurity in terms of the quantity of food required to meet physiological needs. Not only do insects provide calories and nutrients, but they are also cost-effective, easily grown and can be environmentally sustainable when incorporated into a circular production system using organic side streams (136). The establishment of insect production sectors could therefore offer a policy solution to the problem of food insecurity (135). However, several challenges need to be addressed in employing the potential of edible insects to enhance food security. For instance, the nutritional value and health benefits of different insect species should be investigated in more detail to provide the basis for their promotion as a healthy food source (135). In the same way, it is necessary to develop a complete legal framework at the international level that allows the scale of the production process of insects and subsequently the production of derivative products (38).

## Conclusion

In conclusion, it is clear that feeding the growing population is unsustainable over time due to the effects this has on society itself and on the environment. For this reason, today, in different parts of the world, it has been seen that there is a need to create and implement new production systems and new product foods. It has been shown that entomophagy is an alternative with good nutritional qualities, that generates benefits for the environment and people’s lives and that could be a solution to unsustainability. Now, talking about insects, it is concluded that they have a great amount of micro and macronutrients, and that breeding and collection of insects could carry out important livelihood diversification strategies for a social environment improvement and generate business opportunities in developed economies.

In Latin America countries consume most species of insects that belong to the orders *Coleoptera*, *Hymenoptera*, *Lepidoptera*, and *Isoptera*. It has been proven that the two countries that have more species of insects in Latin America are Mexico and Costa Rica and that Mexico has more edible insects compared to the rest of the countries of south and central America.

To scale the production of insects and subsequently the production of derivative products, it is necessary to take into account the type of insect, the diet they require and the optimal conditions for breeding and processing. It was found that temperature and humidity are fundamental conditions to ensure good production and processing of insects. On the other hand, post-harvest process of insects is fundamental to be able to scale the production of these as this includes the operations that would ensure food safety and quality and the processing of the insect into an edible food. So far, blanching has been implemented, which is used to reduce microbiological contamination and inactivate enzymes. It is usually performed prior to food processes such as drying, frying, canning and freeze-drying. In some countries, the mass farming of specific types of insects, like crickets, mealworms and black soldier flies, already exists.

Some Latin America countries do not have a regulatory framework that stables the breeding of insects for the production and marketing of the products, such as Argentina. Countries like Chile, Peru, and Colombia have ventures related with the consumption of food products prepared with insects. Moreover, Brazil does not have ventures because of the lack of information they have about insects, but it is known that indigenous groups tend to consume them, as well as indigenous groups from Colombia. In Colombia, the indigenous people, belonging to the Amazon basin, have the greater proportion of consumption of insects and, even though the alternative of insect consumption is not widely used in this country, there are studies focused on the possibility and formalization of new insect-based products that demonstrate the nutritional and sensory potential of entomophagy.

Facing the current food crisis is a necessity for many countries and, for that reason, it has been proposed as one of the sustainable development goals, which seeks to end all forms of hunger and malnutrition by 2030. It is evident that entomophagy could be considered as a possible solution to this situation because it is less harmful to the environment and insect are nutritious, have high conversion rates and do not require as much space to grow and develop, which could contribute to more people having access to good quality food in all developing countries. Additionally, it would help the growing population to feed itself in the future.

## Author Contributions

SA and MP: conceptualization, methodology, validation, formal analysis, investigation, and writing—original draft. MH-C and AS-C: conceptualization, writing—review and editing, and supervision. All authors contributed to the article and approved the submitted version.

## Conflict of Interest

The authors declare that the research was conducted in the absence of any commercial or financial relationships that could be construed as a potential conflict of interest.

## Publisher’s Note

All claims expressed in this article are solely those of the authors and do not necessarily represent those of their affiliated organizations, or those of the publisher, the editors and the reviewers. Any product that may be evaluated in this article, or claim that may be made by its manufacturer, is not guaranteed or endorsed by the publisher.
